# Informed consent and registry-based research - the case of the Danish circumcision registry

**DOI:** 10.1186/s12910-017-0212-y

**Published:** 2017-09-15

**Authors:** Thomas Ploug, Søren Holm

**Affiliations:** 1 Centre for Applied Ethics and Philosophy of Science, Department of Communication, Aalborg University Copenhagen, A C Meyers Vænge, 2450 København SV, Denmark; 2 University of Manchester, Centre for Social Ethics and Policy, School of Law, Manchester, M13 9PL UK; 3 Center for Medical Ethics, Faculty of Medicine, University of Oslo, Oslo, Norway; 4 Centre for Applied Ethics, Aalborg University, Aalborg, Denmark

**Keywords:** Informed consent, Social pressure, Stigmatization, Medicalization, Discrimination, Polarisation, Health data, Religious circumcision, Meta consent

## Abstract

**Background:**

Research into personal health data holds great potential not only for improved treatment but also for economic growth. In these years many countries are developing policies aimed at facilitating such research often under the banner of ‘big data’. A central point of debate is whether the secondary use of health data requires informed consent if the data is anonymised. In 2013 the Danish Minister of Health established a new register collecting data about all ritual male childhood circumcisions in Denmark. The main purpose of the register was to enable future research into the consequences of ritual circumcision.

**Discussion:**

This article is a study into the case of the Danish Circumcision Registry. We show that such a registry may lead to various forms of harm such as 1) overreaching social pressure, 2) stigmatization, 3) medicalization of a religious practice, 4) discrimination, and 5) polarised research, and that a person may therefore have a strong and legitimate interest in deciding whether or not such data should be collected and/or used in research. This casts doubt on the claim that the requirement of informed consent could and should be waived for all types of secondary research into registries. We finally sketch a new model of informed consent – Meta consent – aimed at striking a balance between the interests in promoting research and at the same time protecting the individual.

**Summary:**

Research participants may have a strong and legitimate interest in deciding whether or not their data should be collected and used for registry-based research whether or not their data is anonymised.

## Background

### The secondary use of health data and informed consent

The secondary use of health data for research is becoming increasingly important, and many countries are developing policies aimed at facilitating such research, sometimes under the banner of ‘big data’ [1, 2]. One of the significant ethical and regulatory discussions in this context is whether the initial registration of the data in the clinical context and/or the later research use should require informed consent from the data subject. The arguments against a requirement for informed consent to data collection and/or research use are strongest in the case where the data collection is necessary for clinical or administrative purposes, i.e. where the later research use is really secondary to another primary purpose for registration; and where the researchers only have access to anonymous (person non-identifiable) data. If the data are truly non-identifiable it can be argued 1) that there is no risk of a harmful infringement of privacy by letting researchers have access to the data, 2) that the persons in question will not otherwise be harmed or have their rights infringed by bona fide health research, 3) that we all have an interest in health research taking place, 4) that a requirement of explicit informed consent would make research impossible; and that there are therefore sufficient grounds for waiving the requirement for informed consent [3–7].

In the following we present and analyse a case study involving the Danish Circumcision Registry, which casts doubt on whether the standard argument for a waiver of informed consent requirements can be extended to all types of health registries and/or all kinds of research using anonymised, secondary data only. We would like to stress that the case of the circumcision registry serves this illustrative purpose in relation to the debate on harm, informed consent and health registries only. We have a longstanding research interest in the secondary use of health data, and this paper grows out of that interest [8–13]. The focus of our analysis is not whether a circumcision registry can be legitimate, but only whether the establishment of such a registry may harm individuals, and whether this creates a sufficient reason for them to be given the possibility to refuse participation. We do not intend to enter into the general debate on male childhood circumcision.^1^

Because our focus is informed consent in relation to the use health data – and not circumcision per se – we proceed from the de facto Danish legal position where ritual circumcision is legal and parents can consent on behalf of their children. They can consent to circumcision, and if a question of consent arises in relation to the collection of health data from children, the parents are also the proxy decision makers. Discussing if parents should have these roles is outside the scope of this paper.

### The Danish circumcision registry

Circumcision of male children is not routinely advocated in Denmark, so such circumcisions are only performed if there is a diagnosed medical problem, e.g. phimosis, or for ritual/religious reasons. Circumcision can only legally be performed by a doctor, or if a doctor is present. In an official report from the Danish Board of Health it has been estimated that there are approximately 15 Jewish and between 1000 and 2000 Muslim ritual circumcisions performed annually in Denmark.

Circumcisions of male children in hospitals have for many years been registered in the National Patient Registry (Landspatientregisteret), and this has allowed some epidemiological research on the effect of circumcision to take place [14]. But since the early 1990s only circumcisions for medical reasons have been performed in Danish public hospitals, since ritual circumcisions have been excluded from coverage in the Danish health care system due to resource constraints. Circumcisions performed outside of hospital, whether or not medical practitioners performed them, have not been registered, and the indication for the circumcisions performed in hospital (i.e. medical or ritual or possibly mixed) has never been registered.

In 2013 the Danish Minister of Health decided to establish a new register aimed at collecting data about all ritual male childhood circumcisions in Denmark. The main purpose of the register was to make future research into the consequences of ritual circumcision possible. In an interview with a Danish newspaper the person administratively responsible for the registry stated “We hope that the new registry (…) will be able to qualify the debate on circumcision. … we have previously not been able to follow up on the potential health related complications following ritual circumcision” [15]. An obligation to report all cases of circumcision was imposed on the general practitioners and practising specialists in a letter from the Ministry of Health in late 2013 [16]. The doctors were required to report the fact that a circumcision had taken place, that it was a ritual circumcision, and the central personal registry (CPR) number of the boy. The CPR number allows linkage to a the vast number of comprehensive health and non-health related registries in Denmark including the main CPR register containing information about family relations and immigration status, The National Patient Registry with information on diagnoses and medical and surgical interventions undertaken at Danish Hospitals, databases of educational attainment, employment status, intelligence at the age of 18 (tested at military conscription) and many other data sources. By linking circumcision to the CPR number the registry would allow for subsequent studies of potential medical and sexual complications following medical and ritual circumcision. The linkage would also allow future identification of and contact with the circumcised boys and men for future research. The circumcision registry did not register the specific religion of the boys, but this can easily be deduced from the timing of the circumcision, the name of the boy, and the immigration status of the parents. Most Danish health registries are run by the semi-independent public institution SSI, and in early 2014 SSI began collecting the data. The collection of data was halted by the new Minister of Health in late July 2015 after it was discovered that the registry did not have the necessary permission to collect the data from the Danish Data Protection Agency (Datatilsynet). In its decision the agency would look at issues of the sensitivity of the data collected, the justification for collecting sensitive data, data security and formal (legal and administrative) aspects relating to the lines of responsibility for data handling. The permission should have been acquired before commencing the collection of data. Thus the registry was in effect without legal foundation in its 19 months of operation under two different Ministers of Health. It was expected that the registry would obtain the required permission and be fully implemented by the end of 2015, but in late 2015 the Ministry of Health decided not to proceed with the registry. The Danish Data Protection Agency never made a final decision concerning the registry. In December 2016 the Ministry of Health issued a regulation that requires medical doctors to report all male circumcisions to the National Patient Registry (Landspatientregisteret) [17]. This will in practice create the same possibilities for research into circumcision as a separate circumcision registry.

In the following we will discuss four aspects of the registry and its use as a basis for circumcision research. We will argue that the establishment of the Danish Circumcision Registry creates the possibility of harm to those who are registered, and that specific features of likely future research creates a situation where persons might legitimately want to opt out of research. The five aspects we analyse are:

1. Social pressure
2. Group stigmatization
3. Medicalization of a religious practice
4. Group discrimination
5. Potential bias in circumcision research as a polarised research field

Our analysis of these five aspects support the conclusion that in this specific instance potential research participants have a strong and legitimate interest in deciding whether or not their data should be collected and whether or not data that have been collected should be used in research.

We will show that the use of anonymised registry data can lead to harms in relation to 1–4 above, and that each of these harms – if they are likely to occur – is sufficient to challenge the assumption that anonymised data can be used without consent. We will further show that 1 and 5 provide autonomy-based reasons to challenge that assumption. By showing that these harms can arise in relation to the use of anonymous data in our case-study we aim to show that it is plausible that they can also arise in other research projects based on anonymous registry data. A general assumption that anonymisation is sufficient to prevent harm and protect participants in registry-based research is therefore false. It is therefore necessary to analyse such research case by case. In specific cases we may find that even though there is identifiable harm to participants, research should still be conducted because it is justified by other important considerations. This, of course, does not add any support to the ‘no harm if anonymised’ assumption prevalent in the literature. It simply shows that we may have overriding reasons.

## Discussion

### The problem of overreaching social pressure

Schoeman in his seminal work on privacy and social freedom directs attention to the simple mechanism that societies make use of social pressure in order to effectively promote certain beliefs and values and thereby secure the continued existence of these societies [18]. Through enforcement mechanisms such as community inclusion or exclusion, and various forms of social gradations involving honour and shame, a society can put pressure on its members in order to secure direction and effectiveness in the promotion and implementation in action of certain beliefs and values that are constitutive of the relevant group of people. In other words social pressure is sometimes necessary to establish a conformity in certain beliefs, values and actions required for the continued existence of a group. Social pressure can, however, sometimes and in some forms become overreaching. That happens when the social pressure towards conformity in beliefs and values makes it impossible for individuals to express and develop their individual identity. Or, in Schoeman’s words, when the social pressure threatens self-expression and self-development. Self-expression and self-development may take various forms, but what is characteristic of both self-expression and self-development is that they engage a person distinctively and they engage a person comprehensively [18]. To somehow express “this is who I am” or “this is who I want to be” is an emotional, cognitive, spiritual and moral engaging act establishing a distinctive individual identity. Social pressure threatens self-expression and self-development by striving towards conformity in beliefs and values. By enforcing beliefs and values upon an individual social pressure may not only cause the erosion of the distinctive self through a process whereby beliefs and values constitutive of the self are substituted by beliefs and values constitutive of a group. By enforcing beliefs and values upon an individual social pressure also restricts the freedom to engage emotionally, cognitively, spiritually and morally in the formation of the self. If self-expression and self-development are to be protected from overreaching social pressure, Schoeman contends, we have to restrict the scope of social pressure as such. This protection may be achieved through granting an individual a social space with freedom from social pressure – a space of privacy. Privacy protects a person against social pressure threatening self-expression. Privacy, defined as restrictions on the physical/observational and informational access to a person, thus limits the access to those beliefs, values, emotions, habits and other traits that may become the target of social pressure and attempt of social control. This conception of privacy clearly implies that the plausibility of a claim to privacy is relative to contexts where there is a risk of social pressure undermining self-expression.

In a wider ethical perspective, the exercise of social control threatens personal autonomy. Personal autonomy may be defined in various ways. Minimally, it may be taken to imply a right to act on preferences that one endorses or at least does not resist without being unduly influenced by others [19, 20]. Social pressure aimed at ‘enforcing’ beliefs and values on the individual seems to leave little room for the individual to endorse or decide not to resist these beliefs and preferences without undue influence. Without going into further details let us simply note that Schoeman’s particular insight is that social pressure may violate something more fundamental than autonomy; it may violate self-expression and self-development as a comprehensive emotional, cognitive, spiritual and moral enterprise. However, self-expression and self-development are clearly forming and acting on preferences that are endorsed by the individual.

There seems to be little doubt that practicing religion in general must be considered an act of self-expression. Religiosity engages the self distinctively and comprehensively. It engages a person cognitively and morally through the formation and justification of beliefs and values but it also engages peoples’ innermost emotions and lead people to form social ties with others of the same belief and partake in various activities of a ritual character [21, 22]. At the same time, religious beliefs are strongly contested in today’s society. They are claimed to be unscientific and irrational, but also to lead to oppression and to be inhuman in various ways [23, 24]. In short, religiosity is a particularly strong form of self-expression with particular potential for becoming the object of social pressure in current societies.

The establishing of a circumcision registry in itself may exercise unacceptable social pressure on religious groups. Establishing the registry has important symbolic effects in that it marks the practice of ritual circumcision as a potential problem that needs investigation, and thereby denormalises the practice. Furthermore, the establishment of the registry is not an isolated event, but an aspect of an ongoing development. A number of Danish medical associations including the Danish Medical Association, the Danish College of General Practitioners, the Danish Surgical Society, and the Danish Paediatric Society have all come out against circumcision unless there is a medical indication [25, 26]. Parents who choose circumcision for religious reasons are thus under suspicion of causing complications to the health of their children, and this creates pressure to critically evaluate their religious practices and potentially give them up.

One may think this pressure justified because circumcision runs counter to some of our most strongly held ethical principles and values, e.g. to avoid causing harm to and mutilation of another person and to secure a valid informed consent before interventions [27–30]. Note very importantly, however, that there is a more general social pressure that is also exercised by such registries, and that is pressure against religiosity as such. Thus the very existence of a public registry with information on peoples’ religious practices may come to affect these practices through general awareness and perhaps even fear of the possibility that the State – public and private researchers – may make one as a religious person the object of not only research but also administrative investigations, but also through fear that one’s religious beliefs may come to play a role for one’s future rights and opportunities in society. That is, the existence of such a registry not only exercises social pressure on the practice of ritual circumcision, but also on religiosity as such.

We are not claiming that unacceptable levels of social pressure will inevitably follow in the wake of establishing a circumcision registry. We are only claiming that establishing a circumcision registry may impact the experienced pressure of the individual, and thus in turn threaten self-expression and self-development related to religious practices. The pressure may infringe self-determination and it may cause harm. This means that the individual has legitimate grounds for resisting both registration and research. More speculatively the establishment of the circumcision registry and the future research use may support stigmatization of the practice of circumcision and those groups in society that practice it.

### The problem of stigmatization

In Erving Goffmans classic interpretation stigmatization is a matter of making a person seem inferior, dangerous and even something less than human by means of attributing the person with a trait that is discreditable to a degree that will result in discrimination. Building on this interpretation, it has been suggested that stigmatization has five constituents [31]. First, the identification and labelling of a difference between people. Second, the association of the relevant difference with negative stereotypes. Third, the segregation of individuals into “us”, the labellers, and “them”, the labelled. Fourth, diminished social status and discrimination. Fifth, an asymmetrical distribution of social, economic and political power that allows for the labelling party to produce segregation and discrimination. According to this theory all five elements are necessary in order for stigmatization to be the case. Several of the elements may, however, vary in degree, and hence strength of stigmatization may vary.

Stigmatization by definition raises ethical issues [9, 32]. Thus the negative stereotyping involved in stigmatization is degrading and dehumanising in nature. It operates by pointing to traits that are seen as deviant and/or shameful, and ultimately, in Goffman’s theory, it involves portraying a person as less worthy than others. As such stigmatization may be claimed to violate a requirement to respect the dignity of others [33]. Stigmatization also per definition involves segregation and discrimination. Finally, there is evidence indicating that stigmatization may cause various psychological harms such as lowered self-esteem, anxiety, depression, and distrust in others [34, 35].

Establishing a registry on ritual or religious circumcision may result in or add to an already existing stigmatization of groups practising circumcision and on religious groups in particular. First, the practice of circumcision is a marked difference between groups in society. In the Danish context circumcision is primarily – if not exclusively – practiced in religious contexts, i.e. specifically in and by the Jewish and Muslim communities. Hence, the difference between the practitioners and non-practitioners is easy to identify and label.

In the Danish public debate circumcision is – partly informed by scientific evidence – claimed to cause various forms of harm to defenceless children ranging from pain during the procedure to complications of sexual life and autism in later life [14, 36–38]. Moreover, circumcision is argued to be a type of intervention that requires a valid informed consent from the patient. This implies that circumcision before the child acquires the competence to consent is a violation of autonomy and of the human rights of the child [39–42]. Thus, strongly present in public discourse is the view that circumcision poses a threat to fundamental values of western societies – it is seen and depicted as inhumane practices. A recent opinion poll shows that 74% of the Danish population think that childhood circumcision should be prohibited [43]. Since circumcision in Denmark is prohibited unless performed by or in the presence of a medical doctor, potential evidence for more complications following ritual or religious circumcision also raises the question if ritual or religious circumcisions are always conducted in conformity with the law. Suspicion of systematic, institutionalised failure to abide by the law may obviously also add to the perceived danger posed by these groups. The public discourse presents circumcision as an inhumane practice, and the establishing of the circumcision registry throws suspicion on religious groups. These are acts of negative stereotyping.

Segregation defined as a separation of individuals into more or less well-defined groups of people labelling and being labelled is already happening in Denmark. The circumcision debate is inextricably linked to debates about the role of especially Muslim immigrants in Danish Society [44, 45]. Establishing a circumcision registry where Muslim boys will be the main group of data subjects potentially makes the Danish public authorities complicit in this labelling of Muslims as deviant.

Whether the general public discourse on circumcision or the specific act of establishing a circumcision registry has or will result in various forms of discrimination in public life is difficult to predict. It is worth noting, however, that the lack of attention to obtaining the required permission from the Danish Data Protection Agency before starting data collection already indicates a lack of interest in the rights of the future data subjects that may be classified as discriminatory.

The asymmetric distribution of power that allows for the labelling party to produce segregation and discrimination is evident in the case of the circumcision. As described above medical societies has taken a stand in the general public debate on circumcision, and the establishment of the registry was ordered by the Minister of Health with the backing of scientific experts. This shows a coming together of expert, professional and political power in this case.

We believe, that all of the above considerations make it likely that circumcised people may already suffer stigmatization to a certain extent but that this stigmatization may be intensified and more strongly directed at religious people by the establishing of the circumcision registry. We do not claim that stigmatization will inevitably follow. Our argument is simply that the mere risk of stigmatization provides an individual with a legitimate reason not to be part of the registry or participate in any future research.

### The problem of medicalization

Zola introduced the concept of medicalization in order to describe the phenomenon of medical concepts being applied to an increasing number of everyday activities. By describing everyday activities in categories of illnesses or in terms of effects on health, these activities become of relevance for the medical profession and may be subjected to medical interventions. As such medicine may increase its role as an institution that exercise social control [46, 47]. The concept of medicalization has also been used to describe a related phenomenon, namely the tendency of people to look at everyday activities from the perspective of medicine. Medicalization in this sense refers to a subtle conceptual change in peoples’ interpretation of events whereby they come to describe events and activities in medical terms [48]. That is, they come to view events or activities in light of the possible effect they may have on their health.

The concept of medicalization has been applied by a number of authors to routine neonatal circumcision as practiced in the USA, [49, 50] and Szasz analysed this practice as one of the many examples of interventions legitimized by the Therapeutic State defined as “…the political order in which social controls are legitimized by the ideology of health” [51].

The ethics of medicalization is complex and cannot be treated exhaustively here [48, 52]. Two general features of medicalization in the latter sense introduced above, however, may make medicalization an ethical problem. First, that the medicalization of events and activities may generate new worries about the future. Viewing events and activities from the perspective of medicine often – if not always – involves identifying and assessing risks to one’s health and wellbeing, and the awareness of such threats may obviously deprive these everyday activities of their pleasure and create new worries. In the case of ritual circumcision medicalization may, for instance engender new worries about sexual function and pleasure. This is obviously a harm to the person in question since circumcision is, for all intents and purposes, an irreversible procedure. Second, there is a further reason to consider medicalization an ethical problem and thus also for the individual to resist medicalization of events and activities, and that is the change in meaning and value per definition involved in the medicalization of events and activities. Medicalization in the latter sense is a matter of people changing their view on things – it is a matter of changing outlook or perspective. Everyday events and activities changes from being endowed with meaning and value independent of the medical context to be events and activities inscribed in a medical framework of meaning and value. Events and activities are interpreted in light of their influence on health and associated with value accordingly.

We believe that establishing a circumcision registry with the purpose of studying potential complications following religious circumcision is likely to lead to or at least further the medicalization of circumcision as religious practice, and that it may undermine the religious meaning and value of these activities. One may obviously for various reasons hold the view that such effects are justified because circumcision runs counter to some of our most strongly held ethical principles and values. Note, however, that reasons for prohibiting circumcision or religious circumcision are not necessarily reasons for medicalization.

It is important to once again note, that we do not claim that medicalization of circumcision as a religious practice will inevitable happen but only that it is likely to be reinforced by establishing of the circumcision registry, and that, most importantly, the possibility of the medicalization of circumcision as a religious practice provides the individual with a reason not to participate in research.

### The problem of discrimination

Apart from the discrimination of individuals in public life conceptually tied to stigmatization, the circumcision registry raises a wider issue of discrimination independent of stigmatization. Let us define discrimination as involving acts and practices that confer harm upon a group of people by treating them differently from relevantly similar groups in society. The very existence of the circumcision registry may be claimed to be discriminatory in this sense because 1) it singles out a minority cultural practice for monitoring of and research into the associated risks among a number of practices associated with relevantly similar risks, and 2) the singling out of this practice may confer the harms of stigmatization and medicalization on group members. Given that we have already argued in favour of 2) let us briefly consider 1).

Many minority and majority cultural practices may be claimed to be associated with risks relevantly similar to those of circumcision. Participation in many sports create a predictable risk of, sometimes permanent physical harm, as does a wide range of other leisure activities, dietary customs etc. Not establishing registries for these practices, while establishing a circumcision registry could therefore be claimed to be inequitable and discriminatory. The inequitability point does, however, cut both ways because it can also be claimed that those participating in these other activities have a claim that their problems should be investigated.

It also has to be taken into account that the establishing of the circumcision registry or any other condition or intervention specific registry is the outcome of a complex political process involving many actors and interests including those of the public, politicians, researchers, research institutions and organisations. A registry is unlikely to be established unless the condition or intervention is perceived as a significant problem by the relevant decision makers since the establishment of a registry requires the expenditure of both political and financial capital, and therefore a decision about how to use and allocate finite resources. The mere fact that there is a registry for one condition or intervention and not for another with the same risk profile is therefore not sufficient to make out a claim of discrimination. Such processes may furthermore lead to decisions on research priorities that are discriminatory in the specified sense without this being intentional.

However, given that the circumcision registry can reasonably be seen to be discriminatory in nature and because of its stated purpose, we believe it provides an individual with a legitimate reason not to be part of the registry or participate in any future research.

### The problem of polarised research

Polarisation of a scientific community in a given field of research obtains when scientists holding radically opposed views are split into groups partly constituted by the opposition to other groups in the field, where this opposition concerns 1) scientific findings and methods, and/or 2) policy advice on the basis of scientific findings [53]. Polarisation may result in the researchers choosing their methods, reporting their findings and offering policy advice with the main aim of promoting the views of their community rather than to advance objective science and provide unbiased advice [53]. In that case we have a conflict of interest between, on the one hand, the general interest of science and the interests of the public and political decision-makers in objective and unbiased science, and, on the other hand, the particular interest of polarised researchers in preserving and advancing the view of a community [54–57]. The polarisation of a scientific community may happen unintentionally. That is, researchers may be honestly convinced of the truth of their views but still act in ways contributing to the polarisation of a scientific community in a given field.

The circumcision research field is polarised to a significant degree. Many researchers studying the effects of childhood circumcision also have strong views about whether circumcision should be promoted, or prohibited. Potential participants in such research arguably have an interest, and putative right, to determine for themselves not only what harms and risks they are willing to expose themselves to in the name of research, but also what kind of research agenda, and which researchers, they wish to align themselves with. For some, a factor in this decision will be the perceived trustworthiness, credibility and scientific objectivity of the researchers involved. To use an analogy, an atheist parent who believes their child’s science teacher was a devout creationist would have a legitimate reason to ask questions about the objectivity of the teaching and seek reassurance about what is being taught and how the teacher manages the conflict between their personal beliefs and the curriculum they must teach. Similarly, a potential participant (who supported circumcision) in a study being run by a researcher who was an outspoken opponent of circumcision has a legitimate reason to ask questions about the objectivity of the study and seek reassurance about how the data is used and interpreted. There may be a perceived conflict of interest between the perceived opposition to circumcision and the ability to independently and objectively evaluate and interpret evidence about its safety, and this provides a person with a legitimate reason to refuse to take part in the research. As such the conflict of interest generated by the polarisation of research is similar to other conflicts of interest. We routinely require informed consent documents to state the funding source for the research, and would not find it inappropriate for someone to decline participation because of a particular funding source. And, like with other conflicts of interest discussions, the premise that needs to be substantiated is not that the conflict of interest will necessarily lead to bias, but only that the conflict of interest may lead to bias. This is the understanding of conflict of interest of the International Council of Medical Journal Editors (ICMJE). In their ‘Form for Disclosure of Potential Conflicts of Interest’ section 5 requires authors to ‘report other relationships or activities that readers could perceive to have influenced, or that give the appearance of potentially influencing, what you wrote in the submitted work’ [58].

### Implications – informed consent as a minimal requirement

In the preceding sections we have argued that the case of the Danish Circumcision Registry illustrates registry-based research that may lead to problematic forms of social pressure, stigmatization, medicalization, discrimination, and that there may be significant conflicts of interest involved. How do we protect the individual against such risks?

Maximal protection of the individual would require, it seems, a prohibition against the establishing of such registries and the research into such registries. If we somehow knew that a registry was established with the purpose of forming the basis for social pressure, stigmatization, and discrimination; or if we knew that a research project had such a purpose, we would have a compelling reason to prohibit the establishment of the registry or the conduct of the research [59]. The mere fact that most or all participants had provided consent would not in itself make the registry or the research legitimate. In the absence of such knowledge this conclusion, however, does not follow. And, it also ignores the positive reasons for conducting research of this kind. After all the research may eventually come to benefit people. And, even if we maintain that the benefits may not always be sufficient to outweigh the costs of the kind described in this paper, it seems that at least sometimes an adequate analysis of this can only be performed retrospectively, i.e. after the research in such registries has been conducted. There is also an argument that only a complete database can form the basis for certain kinds of research. This is an important consideration that has to be weighed against other concerns. Add to that, that we have so far only argued that there is risk of unacceptable forms of social pressure, stigmatization, medicalization, discrimination and of negative consequences of polarisation – not that any of these negative effects will inevitably follow. It seems that we have little ground for ruling out such research entirely.

How then do we ensure then the protection of the individual against the risks associated with this kind of registry-based research? One answer is by requiring informed consent. The requirement of informed consent provides the individual with the opportunity to weigh the reasons for and against participation in registry-based research of the relevant kind, and on this basis provide or refuse consent. We take each of the potential negative aspects – unacceptable social pressure, stigmatization, medicalization, discrimination and biased research – to be sufficient for a requirement of informed consent in a context where they are not only theoretical risks, but have some practical likelihood of actually eventuating.

Several objections may be raised here. First, it could perhaps be argued that we have zeroed in on the single black swan in registry-based research. Most of the current registry-based research does not involve registries and does not have purposes that are likely to have effects similar to that of a ritual circumcision registry, and therefore a general requirement of informed consent would simply be overkill. We believe this objection rests on a too narrow understanding of the triggers of the described problems. To widen the picture, we believe that many forms of research into registries based on data about, for instance, psychiatric disorders, sexually transmitted diseases, ethnicity, and so on, may lead to problematic forms of social pressure, stigmatization, medicalization, and discrimination. And, the problem of polarisation and biased research following from polarisation may happen in any field of research.

Second, one may object that there are alternative ways of protecting the individual. The individual may be protected through anonymization or through a requirement of registry-based research being approved by a research ethics committee. None of these suggestions provide adequate protection of the individual. Thus an individual will not enjoy sufficient protection by simply being anonymised, i.e. by being made non-identifiable. The individual may experience all the negative effects of research using a circumcision registry in spite of being anonymous to the researcher and anybody else. And the legitimate interest in not participating in potentially biased research can never be protected by anonymization. Neither will it provide sufficient protection of the individual to require a research ethics committee to evaluate the risks to the individual. It is not clear that an ethics committee will be able to detect and adequately evaluate the impact on the individual level of the harms potentially suffered from this kind of research. Of all the suggested harms – social pressure, stigmatization, medicalization, and discrimination – it holds that they are highly relative to the individual, i.e. individuals may react and experience these effects very differently. What perspective should the research ethics committee in its evaluation of risks and impacts, the ‘objective view’ of an equanimeous ethnic Danish citizen, or the ‘subjective view’ of a young Muslim man entered into the registry when he was a boy?

Third, one may perhaps make the following “what if” objection: What if the data on religious or ritual circumcision had been routinely collected in the health sector, so that the research into the complications following circumcision would not require the establishing of a new registry, but merely secondary use of data collected for good clinical or administrative research. Would it not have been legitimate to conduct the research without consent in such circumstances? We believe that there is little, if any, ground for distinguishing between the existence of the circumcision registry and research on religious circumcision in the arguments we have advanced. Thus we believe that unacceptable forms of social pressure, stigmatization, the negative effects of medicalization, and biased research may result from research on religious circumcision regardless of the exact way in which the underlying information is originally recorded.

Fourth, one may object that the practical difficulties facing an attempt to obtain informed consent – even if the requirement is somehow restricted to registry-based research projects identified as particularly likely to have the described negative effects – are insurmountable. With today’s technological advancements, we believe, it is possible to find easy and flexible ways of obtaining informed consent using emails, applications for smartphones, or platforms on the Internet. More importantly, however, a requirement of informed consent does not necessarily have to imply a requirement to obtain informed consent for every possible use of personal data (12). A recent model of consent – meta consent [8, 13, 60] – suggests that individuals should be offered the opportunity to design future consent requests for themselves. That is, they should be asked how they would like to provide consent for future use of their health data in research and then given the opportunity to provide an answer using predefined types of consent on predefined types of data. More specifically, it should be possible for an individual to design future consent requests on the basis of types of consent such as:

1. ‘Consent for every use of data’,
2. ‘Consent for broader categories of research on data’,
3. ‘Consent for all research’,
4. ‘Refuse consent to all research’

And for types of data such as e.g.:

A) ‘Electronic Patient Record’ (EPR),
B) ‘Data in health related registries’,
C) ‘Data in non-health related registries’,
D) ‘Biological material’.

The four types of consent may all replace ‘X’ and the four types of data may all replace ‘Y’ in the following statement expressing a consent request: ‘In relation to the future research use of my Y, I wish to be asked for/wish to X’. The resulting consent requests for each individual for the four types of data (A1–4, B1–4, C1–4, D1–4) constitute a meta consent and should be recorded in an official database from which researchers can then automatically generate consent requests based on an individual’s preferences.

Assuming that many individuals would have a high degree of trust in public research institutions and researchers in general and therefore provide a blanket consent for future use of most data, i.e. resulting in a meta consent with the following ordering [A3, B3, C3, D3], an IT based implementation of such a model would imply that less practical difficulties would be faced within a specific research project than models of informed consent requiring a informed consent from every individual for every research project regardless of an individual’s own preference for how and when to provide consent.

## Conclusion

We have in this article argued that potential research participants may have a strong and legitimate interest in deciding whether or not their data should be collected and used for registry-based research such as, for instance, research based on the Danish Circumcision Registry. It is important to note that we have not argued against establishing such registries although some of our considerations could be used in such arguments. It is equally important to note that we have not argued for or against circumcision or ritual circumcision. We have only used the case of the Danish Circumcision Registry to illustrate more general points about research based on secondary use of health and other data.

## Abbreviations

CPR: Central personal registry
SSI: Statens Serum Institut

## Footnotes

1 In the following ‘circumcision’ should be read as ‘male childhood circumcision’ except when explicitly qualified.
